# Racial Disparities in Pediatric Psychiatric Emergencies: A Health Systems Approach

**DOI:** 10.20900/jpbs.20200006

**Published:** 2020-04-13

**Authors:** Abhery Das, Parvati Singh, Tim Bruckner

**Affiliations:** Program in Public Health, University of California, Irvine, CA 92617, USA

**Keywords:** psychiatric care, emergency department, Federally Qualified Health Centers, Community Health Centers, health systems, primary care, mental health care

## Abstract

Less than half of African American youth with severe mental disorders receive psychiatric care. When they do receive care, African American youth use the Emergency Department at higher rates than whites. We examine whether rapid expansion of primary mental health care at Community Health Centers reduces Emergency Department visits for psychiatric care especially among African American youth. Through four studies, we examine (1) the impact of mental health service capacity on the disparity of psychiatric care among African American youth; (2) how Community Health Center mental health visits vary with repeat psychiatric emergency visits; (3) the county-level drivers of the expansion of Community Health Centers; and (4) how Community Health Center expansion affects overall psychiatric emergency care. Results indicate that increased continuity of mental health care at Community Health Centers corresponds with a reduction in racial disparities in youth psychiatric ED visits. In addition, an increase in Community Health Center capacity varies inversely with repeated psychiatric Emergency Department visits and inversely with psychiatric Emergency Department visits overall. And finally, results show an increase in Community Health Center mental health services among counties with greater poverty, lower physician availability, and higher percentage of uninsured. Our studies indicate that expansion of federally-funded primary mental health services affects the overall system of emergency psychiatric care. However, this expansion does not appear to dramatically reduce racial/ethnic disparities in psychiatric emergency department visits.

## INTRODUCTION

About 9 million youth in the U.S. suffer from a severe mental disorder and less than half of them receive care [1]. Undertreatment occurs more often in African American children than white children [2]. African American children also use the Emergency Department (ED) for psychiatric care 11% more than whites and often use the ED for non-urgent conditions [3,4]. Given the current state of EDs in the US, which are often overcrowded and underfunded, this disparity in treatment leads to an increase in health care costs, impedes those who need acute emergency services, and disrupts patient flow [5]. In addition, EDs provide episodic care that may be sub-optimal for psychiatric conditions, compared to non-urgent, routine and primary care settings [5–6]. Little research has looked at the “supply side” of mental health services over time and their potential role in reducing the racial/ethnic disparity in psychiatric ED visits among youth.

Over the past decade, the federal government invested heavily in expanding primary health care across medically underserved communities through expansion of Federally Qualified Health Centers (commonly known as Community Health Centers, or CHCs). CHCs focus on low-income populations and provide comprehensive primary health care for medically underserved communities [7]. They serve over 27 million Americans with African Americans largely represented among patients [8]. Psychiatric visits in CHCs increased by more than 31% from 2006 to 2011 among African American children. Increasing mental health service capacity through CHCs may affect the demand for psychiatric ED visits and reduce the disparity among African American youth psychiatric ED visits. Previous studies examining the expansion of primary care in medically underserved communities, such as Medicaid in Massachusetts and Oregon, show mixed results and fail to show a reduction in disparities [9,10].

**Figure 1.**
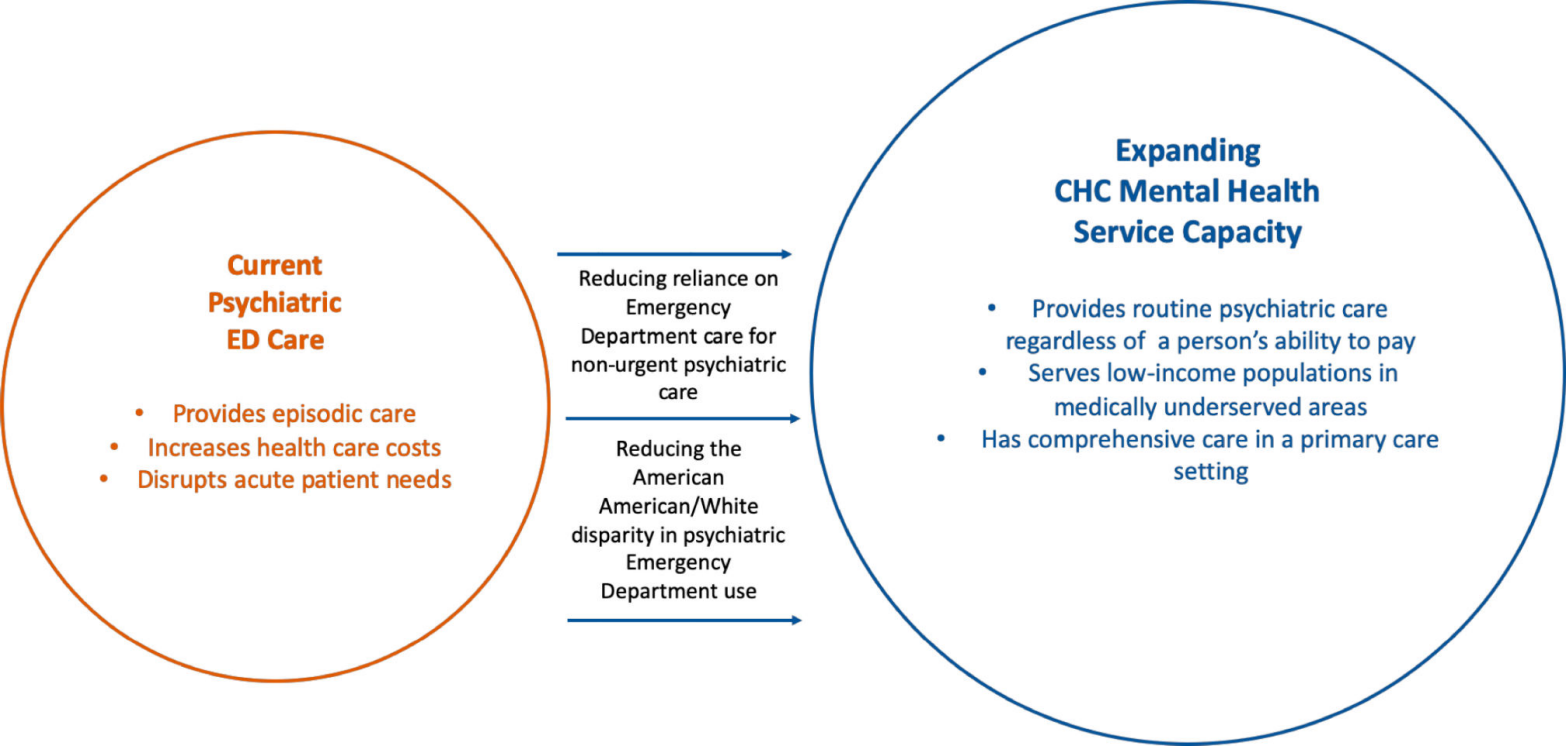
A conceptual framework for examining the role of Community Health Centers on psychiatric Emergency Department Visits.

We address this gap in literature by evaluating, using a longitudinal panel design, whether an increase over time in mental health service capacity (through rapid CHC expansion) corresponds with a reduction in psychiatric ED visits. We focus, in particular, on the African American/white youth disparity on the reliance of psychiatric ED care. Figure 1 outlines our conceptual framework for the four studies we conducted to address this gap. The impact of increasing mental health care capacity for low-income populations and reducing disparities informs policy. The Affordable Care Act invested heavily in CHC expansion. Previous primary care expansion for low-income populations, however, failed to reduce disparities in care [9,10]. We aim to understand the longstanding African American/white disparity in reliance on the ED for psychiatric care and test whether rapid expansion in CHCs reduced this disparity.

## OVERALL PROJECT STRUCTURE

The funded project was a collaboration between scholars from University of California, Irvine, University of California, Berkeley, and Oregon State University. University of California, Irvine served as the lead site for the project under Principal Investigator, Dr. Tim Bruckner.

Dr. Bruckner has published 21 peer-reviewed papers in the area of mental health and substance abuse. He discovered that county-level care provider supply affects racial differences in help-seeking for mental health. He also authored several studies finding that regional changes in the health system and the economy affect emergency psychiatric care, including care among African American children. Dr. Bruckner has a strong background in the application of quasi-experimental methods to identify policy-relevant, regional antecedents of racial/ethnic health disparities. For this project, he supervised the execution of the proposed research and produced the planned publications.

Dr. Lonnie Snowden, co-Investigator, from University of California, Berkeley, is a behavioral health policy and service systems researcher specializing in racial and ethnic disparities in treatment, general health, behavioral access, and quality of care. He identified literature from mental health treatment systems and African American use of mental health services, in addition to assisting and reviewing manuscripts.

Dr. Jangho Yoon, co-Investigator, from Oregon State University, is a mental health economist and has a strong background in applied health econometrics methodology, including causal inference and analysis of healthcare utilization and expenditure data. He assisted with collection, linkage, and analyses of healthcare supply and emergency department data.

Dr. Bharath Chakravarthy, co-Investigator, from University of California, Irvine, is a medical doctor whose expertise lies in understanding mental illness as it presents in the emergency department. He classified relevant diagnostic and procedure codes from Emergency Department databases into meaningful categories.

Parvati Singh, is a PhD candidate in Public Health at University of California, Irvine, who worked as the lead data analyst, conducting all data management, coding, and analysis. She also assisted with manuscript preparation, production, and submission.

## AIMS OF THE GRANT

### Aim 1

Examine the variation over place and time in mental health resources on pediatric psychiatric ED visits and in African American/white disparities. Based on earlier research, we hypothesize that psychiatric ED visits will decline for African American more than white children following rapid increases in CHC mental health treatment capacity for children and/or mental health provider supply. The panel structure of the data permits utilization of region (county) fixed effects that minimize bias due to stable but unmeasured regional factors while controlling for other unobserved spatial and temporal factors.

### Aim 2

Quantify the degree to which the county-level health system affects repeat psychiatric ED visits among youth. An estimated 20% of youth in EDs for psychiatric care return to the ED within a year. Many of these visits are considered non-urgent. We hypothesize that increase in county-level mental health services expansion at FQHCs will correspond with a decline in repeat ED visits.

## DATA AND TIMELINE

We derived our sample of psychiatric ED visits from the State Emergency Department Database (SEDD) and the State Inpatient Database (SID). SEDD and SID are federally sponsored databases made available for purchase under the Healthcare Cost and Utilization Project by the Agency for Healthcare Research and Quality. States contract with HCUP to provide visit-level data from the universe of community hospitals [11,12]. Reliability measures find over 99% of hospital coverage with both databases [13,14]. We purchased visit-level data for Arizona, California, Florida, Kentucky, Massachusetts, Maryland, North Carolina, New Jersey, New York, and Rhode Island from 2006 to 2015. Covering about 30% of the US population, these states represent all four of the US geographic regions [10]. SEDD provides a near-census of all outpatient (same day, “treat-and-release”) ED visits. SID contains comprehensive information on almost all inpatient admissions, including those arising in the ED, among states participating in HCUP. Taken together, SEDD and SID contain nearly 30 million psychiatric ED visits among African American and white children in the state-years included in our research. These datasets contain detailed information on diagnosis (with ICD 9 codes), county identifiers and key visit-level attributes such as age, race, sex and insurance status. SEDD data also permit linkage of repeat ED visits within a calendar year (for select states).

We obtained data on CHCs via the Freedom of Information Act Request from the Uniform Data System (UDS) [15]. In order for CHCs to receive federal funding, the government requires UDS to file reports. CHCs receiving funding from section 330 of the Public Health Service Act must release patient and visit-level summaries for age, race/ethnicity, and mental health services offered [16]. We obtained CHC data for the years 2006–2015 where over 99% of CHCs reported data. UDS data are validated by the U.S. government through data training, consultation, and checks [17]. A total of 310 FQHCs are located within the 9 states we obtained for psychiatric ED visits, providing us with more than 0.4 million annual primary care pediatric psychiatric visits at CHCs.

In addition to the main exposure and outcome variables, we included various relevant covariates in our analyses. Since CHCs serve low-income communities, we included percent of the population living below the poverty line and percent with (or without) health insurance from The Small Area Income and Poverty Estimates Program and the Small Area Health Insurance Estimates datasets [18,19]. We retrieved information on the concentration of physicians from the Area Health Resource File [20]. We also obtained the number of hospital beds, per county-year, from the American Hospital Association [21]. Finally, we included a variable for metropolitan/rural counties as CHC growth may occur unevenly across the US [22].

We submitted the original grant proposal in July 2016. We purchased SEDD and SID data from HCUP, for 9 US states, in July 2017. We obtained CHC data from UDS in May 2017. The University of California, Irvine’s Institutional Review Board (IRB) deemed our research as IRB exempt, therefore no human subjects protocol number was assigned. Since the start of the project in August 2017, we have completed all data management, data analysis, manuscript preparation, and submission. We have published four manuscripts focusing on the aims of the grant proposal: Growth of mental health services at CHCs, Psychiatric ED visits after regional expansion of CHCs, African American/white disparities psychiatric emergencies among youth following rapid expansion of Federally Qualified Health Centers, and Psychiatric-related revisits to the ED following rapid expansion of community mental health services.

## STUDIES

### Study 1: African American/White Disparities Psychiatric Emergencies among Youth following Rapid Expansion of Federally Qualified Health Centers

**Figure 2.**
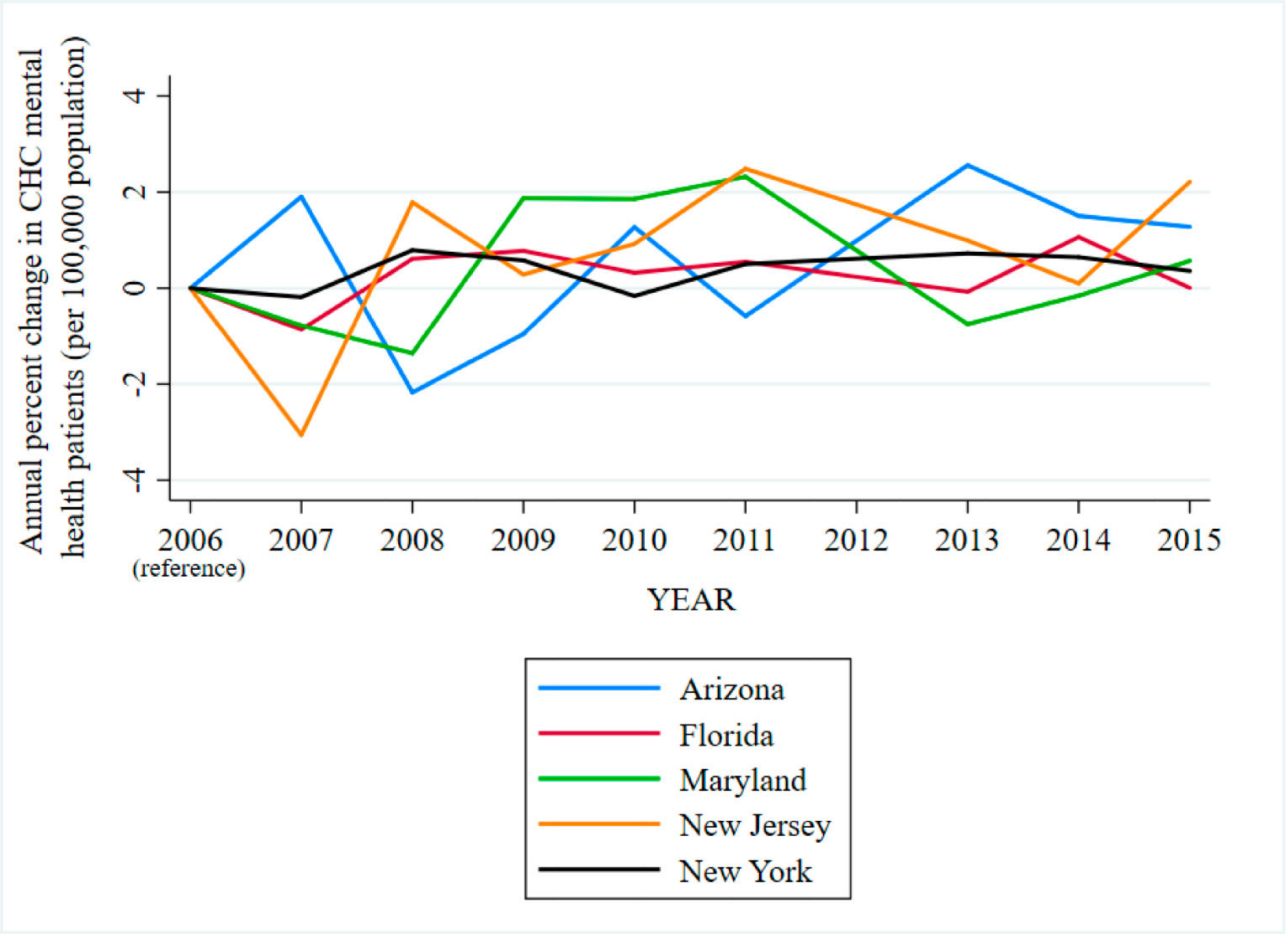
Annual percent change in CHC mental health patients per 100,000 population by year, 2006–2015.

Expansion of low-cost, regional mental health care capacity may reduce disparities in psychiatric emergency Department (ED) visits among African American youth. This population utilizes EDs for psychiatric care more than other race/ethnicities, and their over-reliance on EDs reflects inadequate access to routine, outpatient mental health care [3,4,23]. Community Health Centers (CHCs) provide mental health services and routine follow-up regardless of a person’s ability to pay [24]. Mental health services at CHCs have expanded dramatically over the last decade, with nearly 75% of all CHCs offering mental health care for minority youth populations [25–28]. Figure 2 shows trends in annual percent change in CHC mental health patients per 100,000 population for select states in our sample (Arizona, Florida, Maryland, New Jersey, and New York). In this study, we examined whether expansion of CHC mental health services corresponds with reduction in the odds of a psychiatric ED visit among African American youth, relative to white, across 9 US states, from 2006 to 2011.

We retrieved data on 3 million psychiatric ED visits for African American and white youth from the SEDD and SID for nine US states (Arizona, California, Florida, Massachusetts, Maryland, North Carolina, New Jersey, New York and Rhode Island). We classified psychiatric ED visits based on ICD 9 diagnoses pertaining to mental disorders (Dx1 to Dx25) contained within Clinical Classification Software groups [29]. In keeping with Healthy People 2020, the World Health Organization and contemporary patterns in adolescent growth, we restricted our sample to ED visitors aged 5 to 24 years [30–33]. ED records in SEDD and SID also provide visit-level information on age, sex and insurance status, which we included in our analysis. We obtained these data for the time period of 2006 to 2011 as many states do not report race in SEDD and SID after 2011. We also retrieved county-level information on health system capacity (psychiatric beds and physicians per capita) and socioeconomic attributes (ratio of African American to white youth population, percentage of population in poverty, percentage of uninsured population) from the US Census Population Estimates, the Small Area Income and Poverty Estimates (SAIPE), the Small Area Health Insurance Estimates (SAHIE) and the Area Health Resource File (AHRF) datasets. We obtained county-level data on CHC mental health services from the Uniform Data System.

We specified, as our outcome variable, binary race (1 for African American, 0 for white) of a psychiatric ED visit. We used two measures of CHC health services as our exposures: (i) total number of patients seen at CHCs per 100,000 population per county, and (ii) number of visits per mental health patient at CHCs per county. The first exposure gauges system-level CHC service capacity and the second exposure estimates continuity of mental health care among CHC patients. We log transformed both exposures to reduce the effect of influential outliers.

We conducted logistic regression analysis predicting race as a function of the two (log transformed) CHC exposures (separately), controlling for individual (sex, age, insurance status) and county-level (health system capacity and socioeconomic) attributes. We specified county fixed effects (indicator variables for counties) to account for time-invariant county-specific unobserved factors that may correspond with psychiatric ED visits. Year fixed effects controlled for temporal factors that may affect psychiatric ED visits over our study duration. We examined psychiatric ED visits in SEDD and SID separately as inpatient psychiatric admissions that originate in the ED (SID) reflect greater illness severity relative to outpatient psychiatric ED visits (SEDD). For tests that rejected the null, we estimated the average marginal effect of exposure increments on the odds of an African American psychiatric ED visit, relative to white.

Results from logistic regression analyses show that increase in CHC mental health visits correspond with greater odds of a psychiatric outpatient ED visit (in SEDD) among African American youth, relative to white (Odds Ratio: 1.02, *p* < 0.001). Conversely, increase in continuity of care at CHCs (CHC mental health visits per patient) varies inversely with the odds of a psychiatric outpatient ED visit among African American youth, relative to white (Odds Ratio: 0.96, *p* < 0.001). Application of discovered odds ratios to our data yields about 4200 outpatient psychiatric ED visits “statistically averted” with one percent increase in continuity of care at CHCs. Hypothesis tests among psychiatric inpatient admissions originating in the ED (from SID) fail to reject the null for either exposures.

Taken together, our analytic results suggest that whereas an increase in volume of mental health care supply (i.e., CHC mental health visits) may exacerbate racial disparities in psychiatric ED visits greater continuity of care (CHC mental health visits per mental health patient) through routine follow-up and multiple visits may reduce psychiatric ED reliance among African American youth, relative to white [9]. Integration of mental health and primary care at CHCs may help increase psychiatric care-seeking among vulnerable populations, such as African Americans, and reduce high-cost, preventable ED visits.

Papers & Presentations:

1. Bruckner TA, Singh P, Yoon J, Chakravarthy B, Snowden LR. African American/white disparities in psychiatric emergencies among youth following rapid expansion of Federally Qualified Health Centers. Health Services Research. 2020;55(1):26-34. doi:10.1111/1475-6773.13237 [34].

### Study 2: Psychiatric-Related Revisits to the ED Following Rapid Expansion of Community Mental Health Services

One in five psychiatric patients seen in the ED return for a second ED visit within 6 months [35,36]. Repeat psychiatric visits impose high costs and often do not provide the continuum of care required for treatment and management of psychiatric illnesses [4]. Much research on predictors of repeat psychiatric visits focuses on individual or ED-specific attributes. However, expansion of system-level mental health services may serve in addressing the high burden of psychiatric ED revisits [37]. Regional health care supply through Community Health Centers (CHCs) that provide low-cost, accessible mental health services in primary care settings may reduce repeat ED utilization for psychiatric care. In this study, we examined whether and to what extent increase in mental health services at CHCs correspond with reduction in psychiatric revisits to the ED. We also explored whether this relation varies by the severity or type of psychiatric disorders.

We obtained individual-level data on psychiatric ED visits for 4 US states (California, Florida, North Carolina, New York) from the State Emergency Department Database (SEDD) for the time period of 2006 to 2011 [11]. We identified psychiatric visits in SEDD based on ICD 9 diagnoses for psychiatric disorders. We linked repeat visits per patient, within each calendar year, using the “visitlink” identifier provided by SEDD. We excluded all observations that did not report the “visitlink” identifier [38]. We retrieved data on CHC mental health services from the Uniform Data system. We also obtained county-level information on health care system and socioeconomic attributes for all counties within the 4 study states (CA, FL, NC, NY) from US Census Population Estimates, SAHIE, SAIPE, and AHRF datasets.

We operationalized, as our individual-level outcome, the total count of psychiatric ED visits per patient (per year) less the first visit. Patients with only one ED visit received a revisit count of zero. We specified three county-level exposures that reflect mental health service supply at CHCs: (i) CHC mental health visits (per 100 population), (ii) CHC mental health patients (per 100 population), and (iii) mental health visits per mental health patient at CHCs. Owing to the count nature of our outcome (revisits), we utilized negative binomial regressions to examine the relation between revisits and each of the three exposures (separately). We included patient-level (age, sex, race, insurance status) and county attributes (percentage of population in poverty, percentage of uninsured population, physicians per 100,000 population, number of hospital beds per 100,000 population, percentage of African American population) as controls. Regression methods also controlled for county and year fixed effects. We also conducted negative binomial regression analyses stratified by psychiatric disorder groups (based on illness groups defined in the Clinical Classification Software).

Our final analytic sample comprised about 7.8 million psychiatric ED patients, with 21.3% reporting one or more revisits. The average number of per capita repeat psychiatric ED visits increased from about 0.36 to 0.48 over the study period. Results from negative binomial regression analyses show a 1.4% reduction in psychiatric ED revisits with every one percent increase in CHC mental health visits (per 100 population) (Incidence rate ratio (IRR): 0.986, *p* < 0.001). This relation persists among youth (IRR: 0.983, *p* < 0.001) as well as adults (IRR: 0.97, *p* < 0.001), with slightly stronger association observed among youth. We failed to reject the null for the other two exposures (CHC mental health patients, mental health visits per patient at CHCs). Regression analyses by psychiatric disorder groups finds that increase in CHC mental health visits corresponds with fewer repeat psychiatric ED visits for relatively mild/moderate disorders (e.g., Attention Deficit Disorder, anxiety, mood, alcohol abuse disorders) but not for severe mental illness (e.g., schizophrenia/psychotic disorders). Estimates from average marginal effects suggest that in our sample of 7.8 million psychiatric ED patients, a one percent increase in CHC mental health visits (per 100 population) corresponds with 34,000 fewer psychiatric ED revisits.

An increase in mental health care supply at CHCs may reduce psychiatric ED revisits for mild/moderate mental disorders, but not for severe conditions. By some estimates, heavy utilizers of the ED incur up to six times the cost of care relative to non-repeat ED visitors [39]. Mental health services at CHCs may therefore not only avert excess ED visits but also offer significant cost savings.

Papers & Presentations:

1. Singh P, Chakravarthy B, Yoon J, Snowden L, Bruckner TA. Psychiatric-related Revisits to the Emergency Department Following Rapid Expansion of Community Mental Health Services. Academic Emergency Medicine. 2019;26(12):1336-1345. doi:10.1111/acem.13812 [40].

### Study 3: Rapid Growth of Mental Health Services at Community Health Centers

CHCs provide primary care to medically underserved areas and historically disadvantaged communities [41]. They primarily provide care to persons with Medicaid (38.5% of total) or no health insurance (37.5% of total) [8]. CHCs receive federal funding in the amount of $5 billion per year with $11 billion invested in 2011 [42]. We examined trends in expansion of mental health services at CHCs across the US over a ten-year period from 2006 to 2015.

We retrieved aggregated county-level volume (counts) of CHC patients and visits with a mental health diagnosis including depression and other mood disorders, anxiety disorders including posttraumatic stress disorder, attention deficit and disruptive behavior disorders, in addition to other mental disorders from the Uniform Data System (UDS). We standardized these counts to CHC mental health visits and patients per 1000 population and mental health visits per 1000 county population using population denominators from US Census population estimates [43,44]. Our independent variables, or county-level correlates of CHC mental health expansion, included percent of population living below the poverty line, physician concentration, and metropolitan/rural indicators [19,20,22].

**Figure 3.**
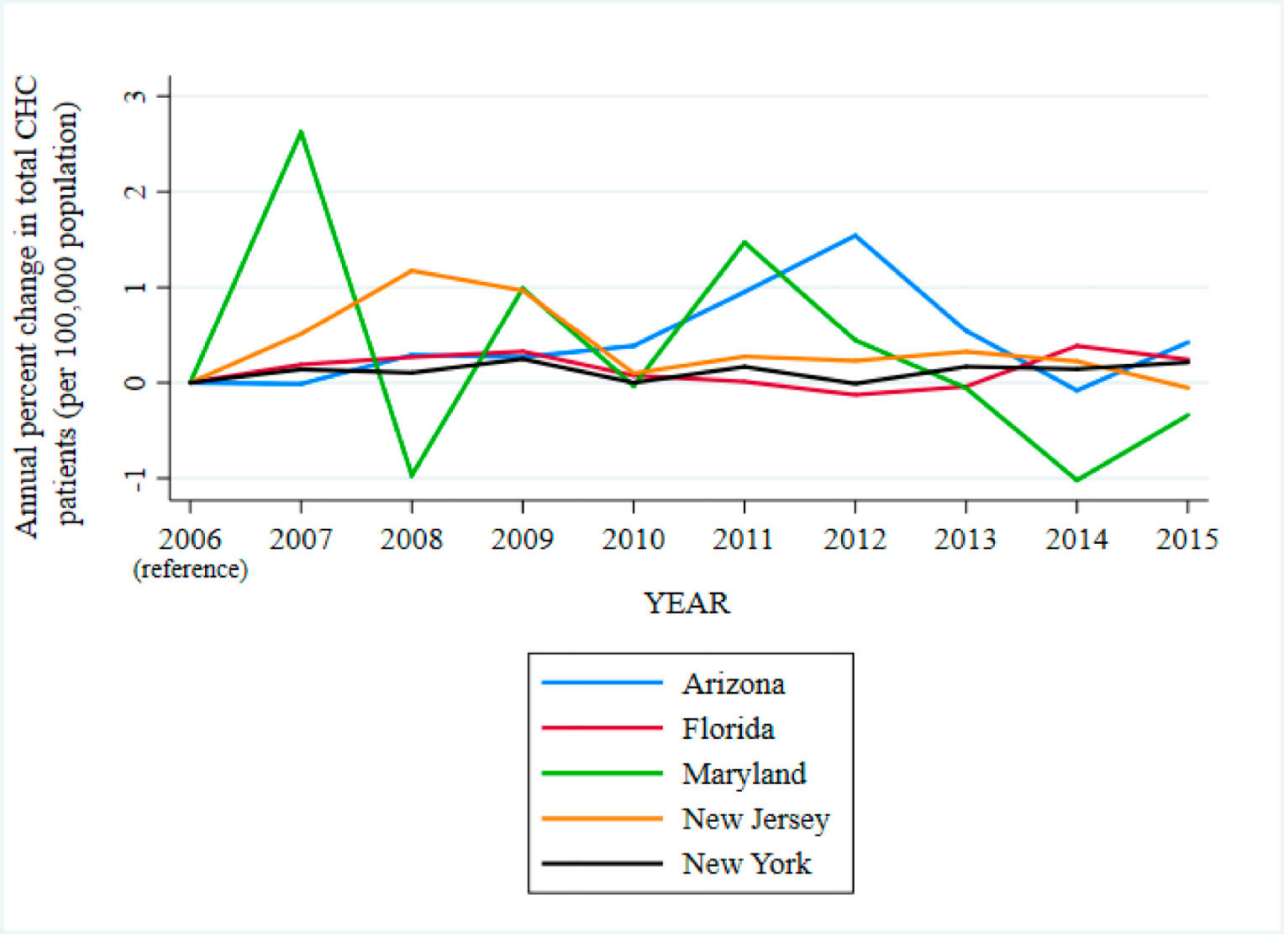
Annual percent change in total CHC patients (per 100,000 population), by state, 2006–2015.

**Figure 4.**
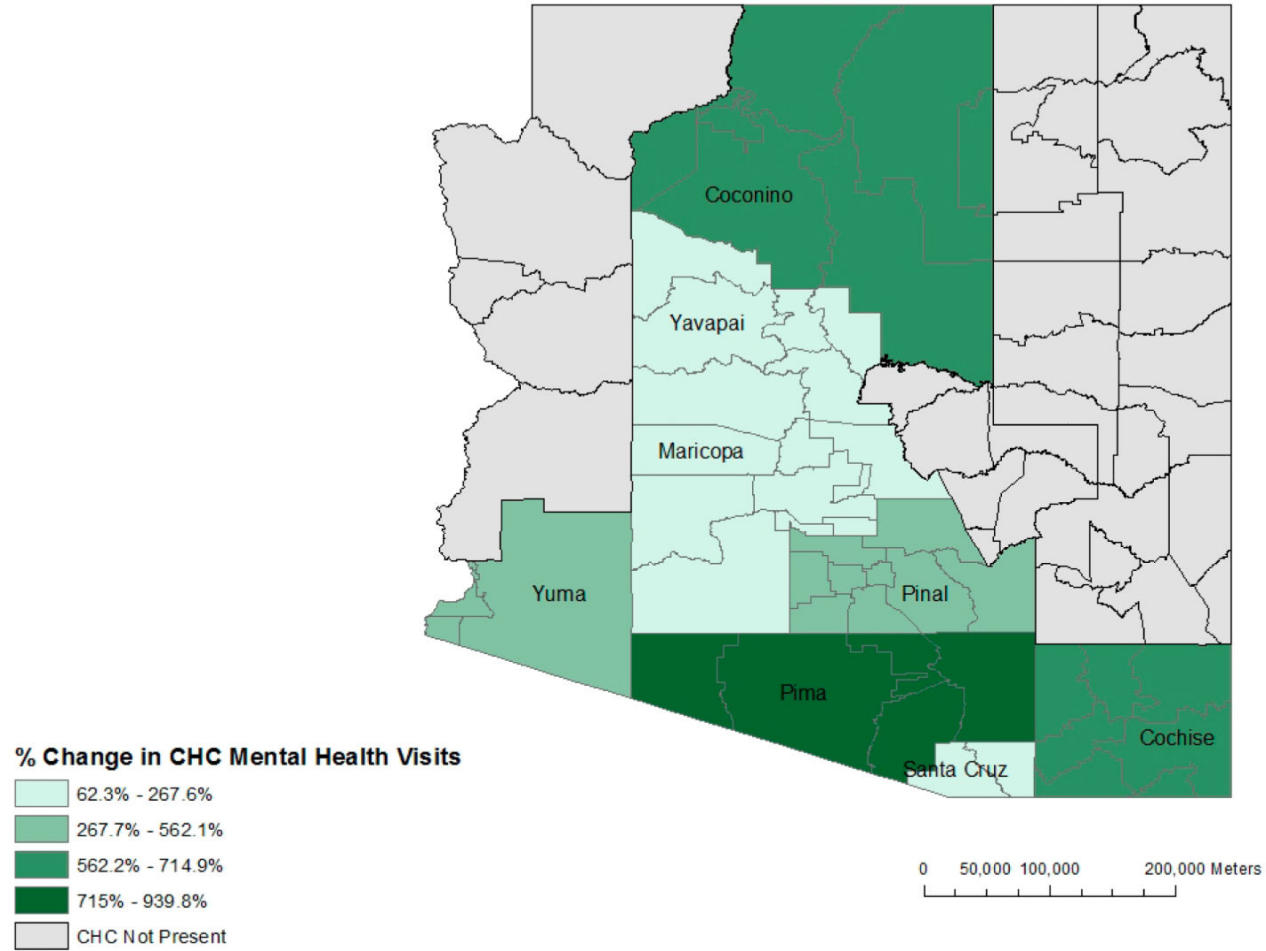
A choropleth of percent change in Community Health Center mental health visits in Arizona by county, from 2006 to 2015.

We found a substantial rise in overall CHC patients from 17.8 to 27.8 million between 2006 and 2015. In Figure 3, we show the annual percent change in total CHC patients per 100,000 population for select states in our sample (Arizona, Florida, Maryland, New Jersey, and New York). CHC mental health visits also increased by 8 to 14% annually from 2007 to 2015. In Figure 4, we show a choropleth of counties in Arizona with percent change in CHC mental health visits from 2006 to 2015. The most populous counties showed the largest increase in mental health visits including, Los Angeles County, New York County, and San Diego County. The top three counties with the greatest percent increase in mental health visits at CHCs consisted of Jefferson Parish (LA), Marathon County (WI), and Yolo County (CA).

Next, we performed longitudinal mixed-effects regression analysis of county-level CHC growth from 2006 to 2011. The reporting of mental health visits and patients at CHCs changed from “primary psychiatric diagnosis only” to “any psychiatric diagnosis” in 2012 [15]. Hence, we examined the time periods of 2006–2011 and 2012–2015 separately. Results show that mental health services at CHCs (patients and visits) expanded in counties with fewer physicians per capita and a greater fraction of white population. Mental health services at CHCs also expanded more in rural, relative to urban counties. Results from 2012 to 2015 analysis show similar results in addition to significant and positive associations for percent poverty and percentage of population with health insurance. Statistical estimates indicate 690 additional mental health visits at CHCs per 100,000 population for every unit increase in percentage of insured population between 2012 and 2015.

We examined county-level correlates of trends in CHC mental health service capacity from 2006 to 2015. We found that expansion of CHC mental health service capacity outpaced the rise in CHC total patient volume over the study period. County-level sociodemographic factors such as percent poverty, percentage of population with health insurance, rurality and supply-side factors (such as lower physician concentration) were positively associated with CHC growth in mental health services. Findings indicate that increase in Medicaid enrollment, after the implementation of the Affordable Care Act, may have contributed to the substantial rise in CHC mental health visits [45]. In addition, the positive association between percentage of white population (in a county) and mental health visits at CHCs sheds light on help-seeking behaviors across race/ethnicity and the potential need for outreach to racial/ethnic minority populations in low-income communities.

Papers & Presentations:

1. Bruckner TA, Singh P, Snowden LR, Yoon J, Chakravarthy B. Rapid Growth of Mental Health Services at Community Health Centers. Adm Policy Ment Health. 2019;46(5):670-677. doi:10.1007/s10488-019-00947-w [28].

### Study 4: Psychiatric Emergency Department Visits after Regional Expansion of Community Health Centers

Psychiatric ED visits account for 5.6 million annual ED visits. Research finds psychiatric ED visits could be avoided if outpatient and community health options enhanced service capacity [46]. Given the U.S. health system of overcrowded yet underfunded EDs, averting psychiatric ED visits could make room for more acute emergency services and cause less disruption to the overall patient flow [5]. CHCs may provide the primary mental health care necessary to deter the reliance on the ED for psychiatric care. Scholars find rates of mental health diagnoses and prescriptions for psychotropic medications increasing and expanding to primary care providers [47]. This indicates that increasing primary care services may increase mental health care capacity. That said, the rapid expansion of primary care at CHCs and the mental health services they provide could deter patients from relying on the ED.

**Figure 5.**
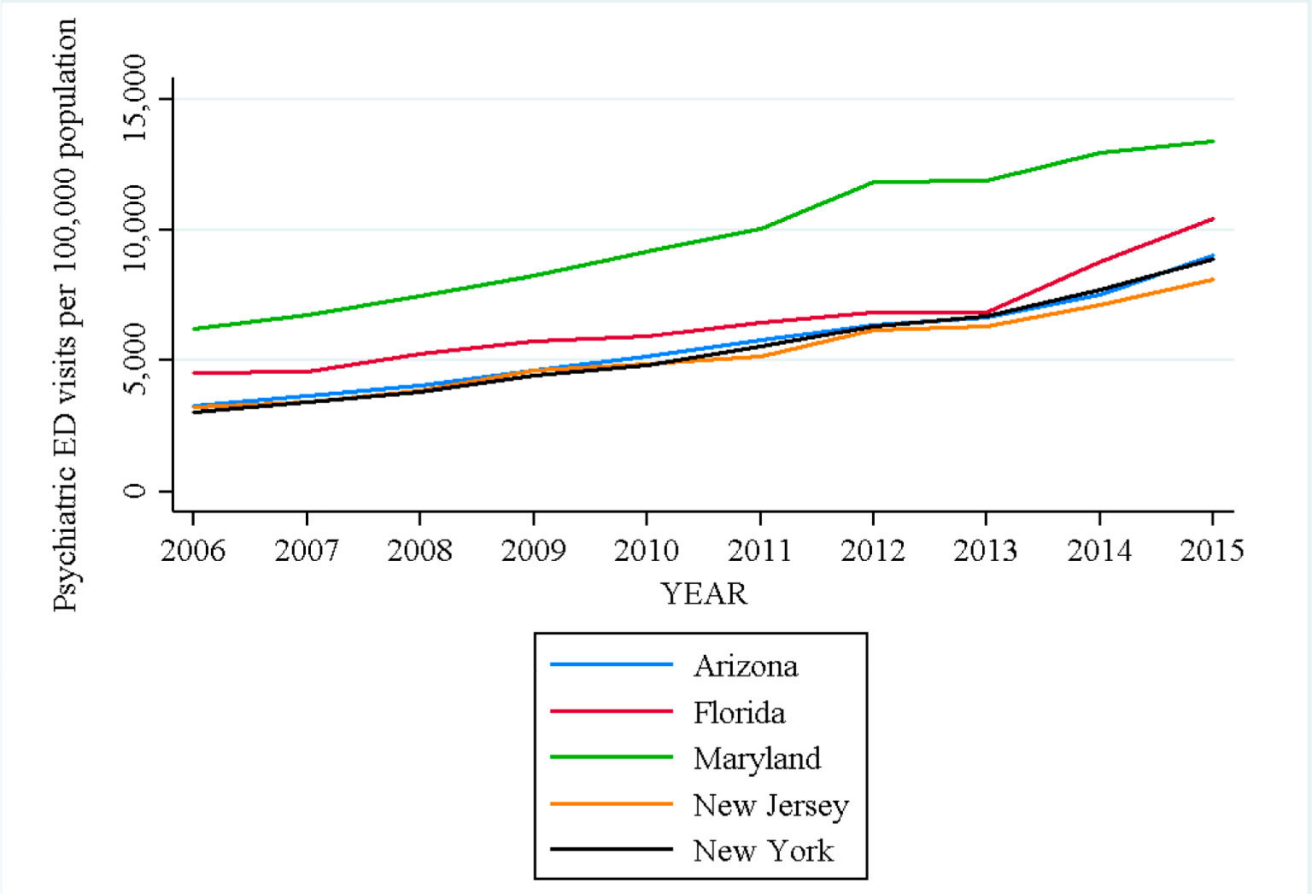
Psychiatric emergency department visits per 100,000 population, Arizona, Florida, Maryland, New Jersey, & New York, 2006–2015.

We measured the association between annual CHC primary mental healthcare visits and annual psychiatric ED visits from 2006 to 2011. We aggregated psychiatric ED visits and patients by county-year and converted them to population prevalence per 100,000 population using US census population estimates [43,44]. Psychiatric ED visits per 100,000 population steadily increased from 2006 to 2011. In Figure 5, we show psychiatric ED visits per 100,000 population for select states in our sample (Arizona, Florida, Maryland, New Jersey, and New York). We aggregated our independent variable, CHC visits, and linked them through county Federal Information Processing Standards codes, yielding us a sample of 745 county-years made up of 143 counties from Arizona, California, Florida, Maryland, Massachusetts, New Jersey, New York, North Carolina, and Rhode Island.

We applied fixed-effects linear regression analysis to account for strong county-level and annual differences by county. We also included covariates to measure mental health profile, help-seeking behavior, and system of care within a county [18–20,44]. From 2006 to 2011, our results showed an average of 5481 psychiatric ED visits per 100,000 population. Total CHC patients average 15,737 per 100,000 population. Psychiatric ED visits rose steadily across the time period as did CHC patients. Fixed-effects regression analysis showed psychiatric ED visits varied inversely with total CHC patients. Our results indicate that an increase in 100 CHC patients corresponds with approximately six fewer psychiatric ED visits.

We observe an inverse relation between psychiatric ED visits and CHC patients across 143 counties from 2006 to 2011. This finding suggests that expansion of primary care through CHCs in low-income communities could divert patients from relying on the ED for psychiatric care. Our results cohere with previous research conducted on the rise in mental health care provided by primary care providers when compared to psychiatrists [48]. Given that our study sample predates the Affordable Care Act, two noteworthy factors may have contributed to the increase in CHC mental health care. The Mental Health Parity and Addiction Equity Act in 2008 allowed those with private insurance to receive comprehensive mental health care [49]. Also, the concept of patient-centered medical homes provided CHCs with incentives to treat chronic conditions comprehensively and to enhance coordination of care [50].

Papers & Presentations:

1. Bruckner TA, Singh P, Chakravarthy B, Snowden L, Yoon J. Psychiatric Emergency Department Visits After Regional Expansion of Community Health Centers. Psychiatr Serv. 2019;70(10):901-906. doi:10.1176/appi.ps.201800553 [51].

## DISCUSSION

**Figure 6.**
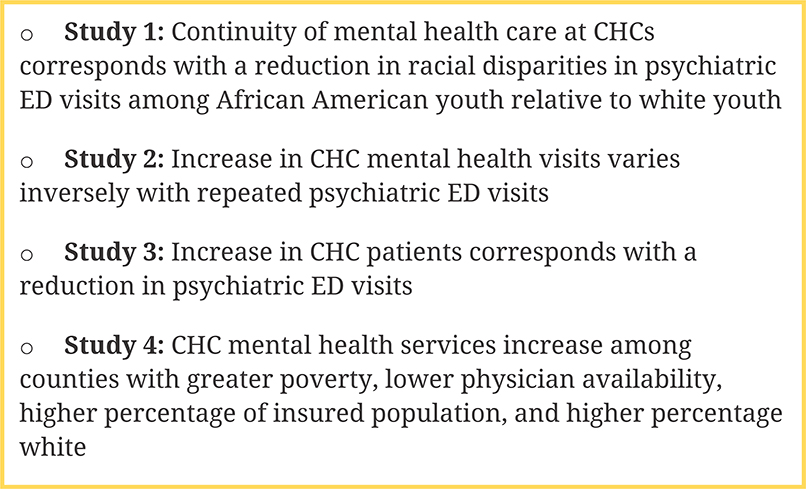
Overall findings from published studies on CHC expansions and psychiatric ED visits.

Under this NIMH funded project, we evaluated whether and to what extent mental health care supply at CHCs corresponds with reduction in psychiatric ED visits across 9 US states. Specifically, we examined whether CHC expansion reduces the racial disparity in psychiatric ED use among African American youth. Figure 6 summarizes the results from our four studies. We find that continuity of mental health care at CHCs corresponds with a reduction in psychiatric ED visits among African American relative to white youth. Examination of trends in CHC mental health services expansion further finds an increase in CHC mental health services among counties with a higher percentage of white population.

Since 2010, federal funding for the expansion of CHCs focused on low-income and medically underserved communities. Given the large representation of African Americans among CHC patients and the substantial increase in CHC psychiatric visits among African American youth (31%), we evaluated whether this expansion would reduce the racial disparity in youth psychiatric ED visits. Results from one of our studies confirms that an increase in mental health visits corresponded with lower odds of a psychiatric ED visit among African American youth. This finding coheres with previous literature among African American youth in California, which discovered greater help-seeking for mental health care following elimination of barriers to low-cost mental health service options [3]. We speculate that CHC expansion may have provided more mental health resources and access to African Americans who otherwise may not have sought mental health care in any setting. The small magnitude of this disparity reduction, however, indicates that CHC expansion on its own does not appear to serve as a cost-effective means of reducing racial disparities in psychiatric ED visits among youth.

Our results also find CHC mental health service capacity increased in counties with more whites, which may reflect greater help-seeking for mental health among white relative to African American populations [52]. Such CHC expansions may, perhaps counterintuitively, exacerbate racial disparities in access to mental health care [52]. Our results cohere with another recent study conducted on the role of CHCs in reducing racial disparities in spatial access to primary care in Philadelphia, PA [53]. Results find that although CHCs improve access to care for neighborhoods with uninsured patients, neighborhoods with higher fractions of racial minorities remain at a higher risk of lower primary care supply [53]. Scholars suggest an explicit focus on racial disparities may be needed regarding placement of CHCs [53].

Our results speak to larger system- and individual-level factors contributing to mental health racial disparities in the US, including health care operations, provider-patient communications, and help-seeking preferences [52,54]. In 2011, The Department of Health and Human Services (HHS) launched an Action Plan to Reduce Racial and Ethnic Health Disparities. Overall goals included steps to recruit health care providers from underserved communities, which has shown to improve health care access for minorities, decrease discrimination, improve patient-provider communication, and improve patient quality and satisfaction outcomes [54,55]. This effort warrants further investigation into whether provider race/ethnicity at CHCs affects African Americans’ demand for mental health care. In addition, the HHS action plan also recommended the evaluation of the Affordable Care Act on racial disparities in health care, as its components offer the potential to address the needs of minority populations [56].

Limitations of our analyses include the inability to link psychiatric ED visits with CHC mental health visits at the individual level owing to aggregate nature of CHC data available. Our analyses, rather, pertain to system-level drivers of psychiatric help-seeking in EDs. We aim to build upon our current work and examine whether higher-quality CHCs, which deliver comprehensive mental health care, show greater effectiveness in reducing psychiatric ED visits among their clients. We also aim to examine whether CHC mental health services supply corresponds with fewer ED visits for suicidal ideation and self-harm and if this relation varies by race/ethnicity. Other projects currently under development include a fine-grained geographic analysis of CHC penetration by zip code, as well as variation in CHC utilization following exogenous ambient and policy-related exposures such as the Affordable Care Act.

## ABBREVIATIONS

ED: emergency department
CHCs: Community Health Centers
FQHCs: Federally Qualified Health Centers
HCUP: Healthcare Cost and Utilization Project
SEDD: State Emergency Department Database
SID: State Inpatient Database
UDS: Uniform Data System
